# Molecular detection of hemoplasmas in rescued black-eared opossums (*Didelphis aurita* Wied-Neuwied, 1826) from southeastern Brazil, with evidence of a novel genotype infecting marsupials

**DOI:** 10.1590/S1984-29612023015

**Published:** 2023-03-27

**Authors:** Ágatha Ferreira Xavier de Oliveira, Ana Cláudia Calchi, Anna Claudia Baumel Mongruel, Anieli Vidal Stocco, Naiara Vidal Stocco, Alexandre Carvalho Costa, Elizabeth Neves Mureb, Jeferson Rocha Pires, Andresa Guimarães, Daniel de Almeida Balthazar, Rosangela Zacarias Machado, Marcos Rogério André, Cristiane Divan Baldani

**Affiliations:** 1 Departamento de Medicina e Cirurgia Veterinária, Universidade Federal Rural do Rio de Janeiro – UFRRJ, Seropédica, RJ, Brasil; 2 Departamento de Patologia, Reprodução e Saúde Única, Faculdade de Ciências Agrárias e Veterinárias – FCAV, Universidade Estadual Paulista – UNESP, Jaboticabal, SP, Brasil; 3 Centro de Recuperação de Animais Silvestres – CRAS, Universidade Estácio de Sá – UNESA, Rio de Janeiro, RJ, Brasil; 4 Instituto Nacional da Mata Atlântica – INMA, Santa Tereza, ES, Brasil

**Keywords:** Hemotropic *Mycoplasma* spp., opossums, hemoplasmosis, wildlife, Hemotrópico, *Mycoplasma* spp., gambás, hemoplasmose, vida selvagem

## Abstract

There is a growing concern about the participation of wild hosts and reservoirs in the epidemiology of several pathogens, particularly within the context of environmental changes and the expansion of the One Health concept. The aim of this study was to investigate the presence of hemoplasmas in opossums rescued from the metropolitan region of Rio de Janeiro state, Brazil. Blood samples were collected from 15 *Didelphis aurita* and subjected to DNA extraction and PCR using primers for the 16S rRNA and 23S rRNA genes. Physical examination and hematological analysis were also performed. Three out of 15 opossums tested positive for hemotropic *Mycoplasma* spp. by PCR and showed hematological alterations such as anemia and leukocytosis. Clinical signs were non-specific and associated to traumatic lesions. The phylogenetic analysis indicated that the hemoplasma detected was positioned between '*Ca*. Mycoplasma haemodidelphis' detected in *D. virginiana* from North American and hemoplasmas recently detected in *D. aurita* from the state of Minas Gerais, Brazil. This study indicates the existence of hemoplasma infections in *D. aurita* from the metropolitan region of Rio de Janeiro, and reinforce the need for new epidemiological inquiries to clarify the participation of these in the dynamics of circulation of tick-borne pathogens.

## Resumo

Há uma crescente preocupação com a participação de hospedeiros e reservatórios silvestres na epidemiologia de diversos patógenos, principalmente no contexto das mudanças ambientais e da expansão do conceito "One Health". O objetivo deste estudo foi investigar a presença de hemoplasmas em gambás resgatados da região metropolitana do estado do Rio de Janeiro, Brasil. Amostras de sangue foram coletadas de 15 *Didelphis aurita* e submetidas à extração de DNA e PCR utilizando-se "primers" para os genes 16S rRNA e 23S rRNA. O exame físico e a análise hematológica também foram realizados. Três dos 15 gambás testaram positivo para *Mycoplasma* spp. hemotrópico por PCR. Os sinais clínicos eram inespecíficos e associados a lesões traumáticas. Anemia e leucocitose foram detectadas em animais positivos. A análise filogenética indicou que o hemoplasma detectado estava posicionado entre *'Ca.* Mycoplasma haemodidelphis' detectado em *D. virginiana* da América do Norte e hemoplasmas recentemente detectados em *D. aurita* do estado de Minas Gerais, Brasil. Este estudo indica a existência de infecções por hemoplasmas em *D. aurita,* da região metropolitana do Rio de Janeiro, e reforça a necessidade de novos inquéritos epidemiológicos, para esclarecer a participação destes na dinâmica de circulação de patógenos transmitidos por carrapatos.

## Introduction

*Didelphis aurita* Wied-Neuwied, 1826, popularly known as black-eared opossum, is one of the most common species of marsupials in the Neotropics, inhabited from northeastern Brazil to northeastern Argentina. It is found mainly in forested areas with low altitudes (Hershkovitz, 1969) and, despite being omnivorous, their food is predominantly composed of insects and other invertebrates (Cáceres, 2004).

Opossums (*Didelphis* sp.) are among the most ecologically important wild mammals in the Americas and play a key role in insect control and seed dispersal (Bezerra-Santos et al., 2021). These mammals are also classified as synanthropic animals since they have adapted to urban environments, as a result of environmental changes that occurred during the intensification of the urbanization process (Gonçalves et al., 2021). These marsupials are suggested as possible reservoirs of zoonotic protozoans, such as *Leishmania* sp. and *Trypanosoma* sp., and once inserted in unusual ecological niches, contribute to changes in the dynamics of transmission of these and other pathogens previously restricted to wild environments (Price et al., 2019; Carreira et al., 2012).

Hemotropic mycoplasmas (hemoplasmas) are Gram-negative pleomorphic bacteria that adhere to the erythrocyte surface of mammals, causing hemolytic anemia of several degrees (Messick, 2004). The mechanisms of natural transmission in animals have not yet been fully elucidated. To date, hemoplasmas known to infect *Didelphis* are '*Candidatus* Mycoplasma haemoalbiventris', described in white-eared opossums (*D. albiventris*) in the south and central-west regions of Brazil (Massini et al., 2019; Gonçalves et al., 2020; Pontarolo et al., 2021; Oliveira et al., 2021) and black-eared opossums (*D. aurita*) in southeastern Brazil (Orozco et al., 2022). Additionally, ‘*Candidatus* Mycoplasma haemodidelphis’ have been detected in the Virginia opossum (*D. virginiana*) from USA (Messick et al., 2002).

Mycoplasma infections are described in many species of wild mammals in Brazil, such as vampire bats (Correia dos Santos et al., 2020), rodents (Gonçalves et al., 2015), among others. The prevalence of parasitic inclusions in rodent and marsupial red blood cells was 17.4% (Silva et al., 2007). In molecular assays, the prevalence of hemoplasma infection observed in free-living capybaras and monkeys from Brazilian Atlantic Forest was higher than direct observation, with values of 76.47% and 66.7%, respectively (Vieira et al., 2021; Ramalho et al., 2017). Distinct population characteristics in addition to the different diagnostic techniques employed may influence the frequency of mycoplasma infection in different Brazilian regions (Biondo et al., 2009).

Considering the proximity of *Didelphis* sp. to urban environments, as well as their potential to harbor infectious agents of importance in human and animal health, the present study aimed to investigate the occurrence and molecular identity of hemoplasmas in peripheral blood samples from recued black-eared opossums (*Didelphis aurita*) from the metropolitan region of Rio de Janeiro, southeastern Brazil.

## Material and Methods

### Sampling

From August 2020 to July 2021, a total of 15 adult (ten females and five males) black-eared-opossums (*Didelphis aurita*) selected by non-probability convenience, were received at two rescue centers of the metropolitan region of Rio de Janeiro: “Centro de Triagem de Animais Silvestres” (CETAS-RJ) and “Centro de Reabilitação de Animais Silvestres” at Universidade Estácio de Sá (CRAS UNESA-RJ), state of Rio de Janeiro, southeastern Brazil. CETAS-RJ is located in Seropédica municipality (22◦45014” S and 42◦52055” W), which is surrounded by Atlantic forest and where there is a Nature Conservation Unit (Mário Xavier National Forest) of 43 hectares. On the other hand, CRAS UNESA-RJ is located in an urbanized area. Animals rescued in the regions nearby were directed to these sites.

Out of the 15 animals rescued, seven (46,67%) were adult females, three (20%) sub-adult females, three (20%) adult males and two (13,33%) sub-adult males. Blood samples were collected by venipuncture of the tail vein and preserved in ethylenediaminetetraacetic acid (EDTA)-treated tubes. Subsequently, blood samples were used for hematological analysis and an aliquot was stored at -20 °C until further use for molecular investigation.

The animals were clinically examined by general and specific clinical examinations (Nascimento & Horta, 2014). Visual inspection for ectoparasites (ticks and fleas) was also performed and classification carried out according to previously described morphological taxonomic keys (Linardi & Santos, 2012).

### Hematological analysis

Hematological parameters were determined manually as described by Jain (1993). Packed cell volume (PCV) was determined by the microhematocrit technique and total plasma protein (TPP) concentration determined by using a refractometer. Differential leukocyte counts were performed manually on Diff Quick-stained (Laborclin, Brazil) thin blood films, using an optical microscope, with immersion objective lens magnification of 1000x. Blood count data were expressed through the mean and standard deviation (SD). For comparison of data, reference values were considered as determined by Casagrande et al. (2009) and Orozco et al. (2022).

### DNA extraction and quality assessment

DNA was extracted from 200 µL of blood samples using the DNeasy® Blood&Tissue Kit (Qiagen®, Valencia, California - USA) according to the manufacturer's recommendations. To avoid false-negative results and monitor DNA extraction, an internal control PCR for all samples based on the endogenous mammalian gene *gapdh* was performed (Birkenheuer et al., 2003).

### Molecular detection and characterization of hemoplasmas

Opossums’ blood DNA samples were screened for hemoplasmas DNA using a PCR protocol targeting a fragment (900 bp) of the 16S rRNA gene (Maggi et al., 2013a). Subsequently, the positive samples were submitted to a semi-nested PCR assay based on a 1107 bp fragment of the 16S rRNA gene (Di Cataldo et al., 2020), as well as to a PCR targeting fragments of the 23S rRNA (800 bp) (Mongruel et al., 2020) and *RNAse P* (165 bp) (Maggi et al., 2013b) genes (Table 1). Only amplicons obtained from the semi-nested PCR based on the 16S rRNA gene and from PCR based on the 23S rRNA gene were sequenced. The assays were performed using 5 μL of the DNA samples in a mixture containing 1.5U Platinum Taq DNA Polymerase (Invitrogen, Carlsbad, California, USA), PCR buffer (PCR buffer 10 X – 100 nM Tris-HCl, pH 9.0, 500 mM KCl), 0.8 mM deoxynucleotides (dATP, dTTP, dCTP, and dGTP) (Invitrogen, Carlsbad, California, United States), 1.0 mM of Magnesium chloride (Invitrogen, Carlsbad, CA, United States), 0.3 μM of each primer (Invitrogen), and sterile ultrapure water (Invitrogen) q.s. 25 μL. In the semi-nested PCR assay, 1 μL of the amplified product from the first PCR reaction was used as the template in the second reaction. *Mycoplasma suis* DNA sample obtained from naturally infected pigs (Gatto et al., 2019) and ultrapure sterile water were used as positive and negative controls, respectively, in all PCR assays for hemoplasmas.

**Table 1:** Description of primers, amplicon sizes and termal sequences used in conventional and nested PCR assays.

**Agents**	**Primer sequences**	**Size (bp)**	**Thermal sequences**	**References**
Hemoplasmas (16S rRNA gene)		900	94°C for 2 minutes	Maggi et al. (2013a)
- HemMycop16S-41s	5’-GYATGCMTAAYACATGCAAGTCGARCG-3’		55 cycles: 94°C for 15 seconds, 68°C for 15 seconds and 72°C for 18 seconds	
- HemMyco16S-938as	5’-CTCCACCACTTGTTCAGGTCCCCGTC-3’		72ºC for 1 minute	
Hemoplasmas (16S rRNA gene) External primers		1428	95°C for 5 minutes	Di Cataldo et al. (2020)
- HemoF1	5’-AGAGTTTGATCCTGGCTCAG-3’		35 cycles: 95°C for 30 seconds, 57°C for 30 seconds and 72°C for 1 minute	
- HemoR2 Internal primers	5’-TACCTTGTTACGACTTAACT-3’		72ºC for 10 minutes	
- HemoF2		1107		
- HemoR2	5’-ATATTCCTACGGGAAGCAGC-3’			
Hemoplasmas (23S rRNA gene)		800	94°C for 3 minutes	Mongruel et al. (2020)
-23S_HAEMO_F	5'- TGAGGGAAAGAGCCCAGAC-3'		35 cycles: 94°C for 30 seconds, 54°C for 30 seconds and 72°C for 1 minute	
-23S_HAEMO_R	5’- GGACAGAATTTACCTGACAAGG-3'		72ºC for 10 minutes	
Hemoplasmas (*RNAse P* gene)		165	95°C for 2 minutes	Maggi et al. (2013b)
HemoMyco*RNaseP*30S	5’- GATKGTGYGAGYATATAAAAAATAAARCTCRA C-3’		55 cycles: 94°C for 15 seconds, 59°C for 15 seconds and 78°C for 18 seconds	
HemoMyco*RNaseP*200 as	5’-GMGGRGTTTACCGCGTTTCAC-3’		72ºC for 30 seconds	

### Agarose gel electrophoresis

The products obtained in PCR assays were separated by horizontal electrophoresis on a 1% agarose gel stained with ethidium bromide (Life Technologies™, Carlsbad, CA, USA) in TEB running buffer pH 8.0 (44.58 M Tris-base; 0, 44 M boric acid; 12.49 mM EDTA) at 100 V/150 mA for 50 min. To determine the size of amplified products, a 100 base pair molecular weight marker (Life Technologies^®^) was used. The gels were imaged under ultraviolet light (ChemiDoc MP Imaging System, Bio Rad™, Hercules, CA, USA) using the Image Lab Software v4.1 (Biorad, Hercules, CA, USA).

### Purification of PCR amplified products and sequencing

The amplified products were purified using Wizard® SV Gel kit and PCR Clean-Up System (Promega), according to the manufacturer's recommendations. The sequencing was carried out using the dideoxynucleotide chain termination method (Sanger et al., 1977), conducted in the ABI PRISM 3700 DNA Analyzer sequencer (Applied Biosystems^®^) at the Centro de Recursos Biológicos e Biologia Genômica (CREBIO - Faculdade de Ciências Agrárias e Veterinárias – UNESP/Campus de Jaboticabal).

### Phylogenetic analysis

The sequences obtained were submitted to a quality-screening test using Phred-Phrap software (version 23) (Ewing et al., 1998) to evaluate the quality of the electropherograms and to obtain the consensus sequences from the alignment of the sense and antisense sequences. The BLASTn program (Altschul et al., 1990) was used to compare the obtained nucleotide sequences with previously deposited sequences in the GenBank database (Benson et al., 2005). The sequences saved in “FASTA” format were aligned with other homologous sequences of each agent retrieved from the database (Genbank), using the Mafft software (Katoh & Standley, 2013) and edited via Bioedit v. 7.0.5.3 (Hall, 1999). W-IQ-Tree software was used for the choice of the evolutionary model following Akaike criterion and for phylogenetic analysis by the Maximum Likelihood method (Trifinopoulos et al., 2016), while clade support indices were evaluated through bootstrap analyses (Felsenstein, 1985) of 1000 repetitions. The editing of phylogenetic trees as well as rooting (via outgroup) were performed using the Treegraph 2.0.56-381 beta software (Stöver & Müller, 2010).

### Genetic diversity and genotype network

The genetic diversity analysis for the 16S rRNA gene were performed with the alignment of hemotropic *Mycoplasma* sp. sequences detected in opossums from Brazil and worldwide. This alignment was used to calculate the nucleotide diversity (π), polymorphism level (diversity of haplotypes [Dh], number of haplotypes [h], and the average number of nucleotide differences [K]), using DnaSP v5 software (Librado & Rozas, 2009). The Genotype network was constructed in PopART, using the TCS inference method (Clement et al., 2000; Huson & Bryant, 2006).

## Results

At time of sampling, animals were not infested with ticks and only three (20%) black-eared opossums presented fleas (*Ctenocephalides felis felis*). The mean and range values obtained in hematological analysis of black-eared opossums (*D. aurita*) are summarized in Table 2. Ten (66%) animals presented hematocrit, hemoglobin and red blood cell counts below the mean values ​​reported by Casagrande et al. (2009), and they were considered anemic. Regarding the leukometry values, 73.3% of the animals presented values ​​higher than the average reported by Casagrande et al. (2009). A total of ten blackeared opossums presented different clinical alterations: ophthalmologic lesions, electric shock burns, skin lesions, lymphadenomegaly, traumatic lesions, parasitic infestations, hemorrhages and neurological disorders.

**Table 2:** Mean and Standard Deviation (SD) of the blood count (blood count) of black-eared opossums (*Didelphis aurita*) (n = 15) and comparative values proposed by Casagrande et al. (2009) and Orozco et al. (2022).

		**Erythrocyte**		
			**References (Mean ± SD)**	
**Analyte**	**Mean ± SD**	**Range (Min-Max)**	**Casagrande et al. (2009)**	**Orozco et al. (2022)**
Red blood cells (×10^6^/µL)	4.48 ± 1.98	2.19 - 10.30	4.30 ± 1.55	4.31 ± 1.06
Hemoglobin (d/dL)	8.68 ± 2.82	3.09 - 14.70	10.96 ± 3.44	11.77 ± 1.99
PCV (%)	29.73 ± 7.78	18.00 - 50.00	31.85 ± 8.00	38.15 ± 6.47
Total protein (g/dL)	6.41 ± 1.29	4.00 - 8.00	8.49 ±1.04	7.41 ± 0.85
MVC (fl)	76.03 ± 4.87	70.40 - 83.50	78.13 ± 18.13	84.00 ± 8.17
MCHC (%)	29.72 ± 1.71	25.20 - 31.50	64.59 ± 8.12	30.91 ± 1.84
		**Leukocytes**		
**Analyte**	**Mean ± SD**	**Range (Min-Max)**		
Leukocytes (/µl)	12080.00 ± 6308.97	1700 – 21800	8205 ± 4950	14596.67 ± 6200.58
Neutrophils (/µl)	6281.60 ± 3280.66	884 - 11336	2761 ± 2966	6755.37 ± 3614.52
Lymphocytes (/µl)	4865.13 ± 2420.34	583 - 9932	3653 ± 2431	5306.87 ± 2499.46
Monocytes (/µl)	304.13 ± 388.04	45 - 1593	363.2 ± 308.7	416.43 ± 493.10
Eosinophils (/µl)	1425.46 ± 1251.52	218 - 4180	1362 ± 1114	1790.77 ± 1603.63
Basophils (/µl)	105.26 ± 148.70	0 - 452	65.90 ± 127.0	202.30 ± 246.69
		**Platelets**		
**Analyte**	**Mean ± SD**	**Range (Min-Max)**		
Platelets (×10^3^/µL)	408.533 ± 274.713	199.000 – 1.000.000	-	284.96 ± 173.23

The mammalian endogenous *gapdh* gene was consistently amplified in all samples. Hemoplasma DNA was detected in 20% (3/15) of the blood samples tested by PCR based on 16S rRNA gene in this study, which originated from two adult females and one adult male from CETAS-RJ. Additionally, all three blood samples were positive in PCR assays based on the 16S rRNA (fragment of approximately 1,107 bp) and 23S rRNA genes. No samples were positive for the *RnaseP* gene.

The three PCR positive black-eared opossums presented traumatic lesions on physical examination, as well as anemia and leucocytosis.

Despite three rescued animals were positive, only two samples of 16S rRNA and 23S rRNA were successfully sequenced. The BLASTn analyses showed that the sequences obtained in the present study for 16S rRNA gene were identical to the sequence of ‘*Candidatus* Mycoplasma haemoalbiventris’ detected in black-eared opossum from Brazil. Whereas, the 23S rRNA sequences had approximately 99% identity with ‘*Candidatus* Mycoplasma haemoalbiventris detected in white-eared opossum (Table 3).

**Table 3:** nBLAST analysis results for the obtained hemoplasma 16S and 23S rRNA sequences from black-eard opossums (*Didelphis aurita*) blood samples.

**Species Identify (ID) /localization**	**Target gene**	**Sequence length (bp)**	**Query coverage (%)**	**E-Value**	**Identity (%)**	**GenBank accession numbers**
*Didelphis aurita* 7S /CETAS-RJ	16S rRNA	813	100%	0	100%	‘*Ca.* Mycoplasma haemoalbiventris’ sp. - *Didelphis aurita* from Brazil (OP279617)
*Didelphis aurita* 7S /CETAS-RJ	23S rRNA	585	99%	0	99.49%	‘*Ca.* Mycoplasma haemoalbiventris’ - *Didelphis albiventris* from Brazil (MN442084)
*Didelphis aurita* 22S /CETAS-RJ	16S rRNA	746	100%	0	100%	‘*Ca.* Mycoplasma haemoalbiventris’ sp. - *Didelphis aurita* from Brazil (OP279617)
*Didelphis aurita* 22S /CETAS-RJ	23S rRNA	546	100%	0	99,45%	‘*Ca.* Mycoplasma haemoalbiventris’ - *Didelphis albiventris* from Brazil (MN442084)

All 16S rRNA sequences obtained were deposited in Genbank under the following accession numbers: ON921333, ON921332. In addition, the 23S rRNA sequences obtained were deposited under the following accession numbers: ON920560, ON920559.

**Figure 1:**
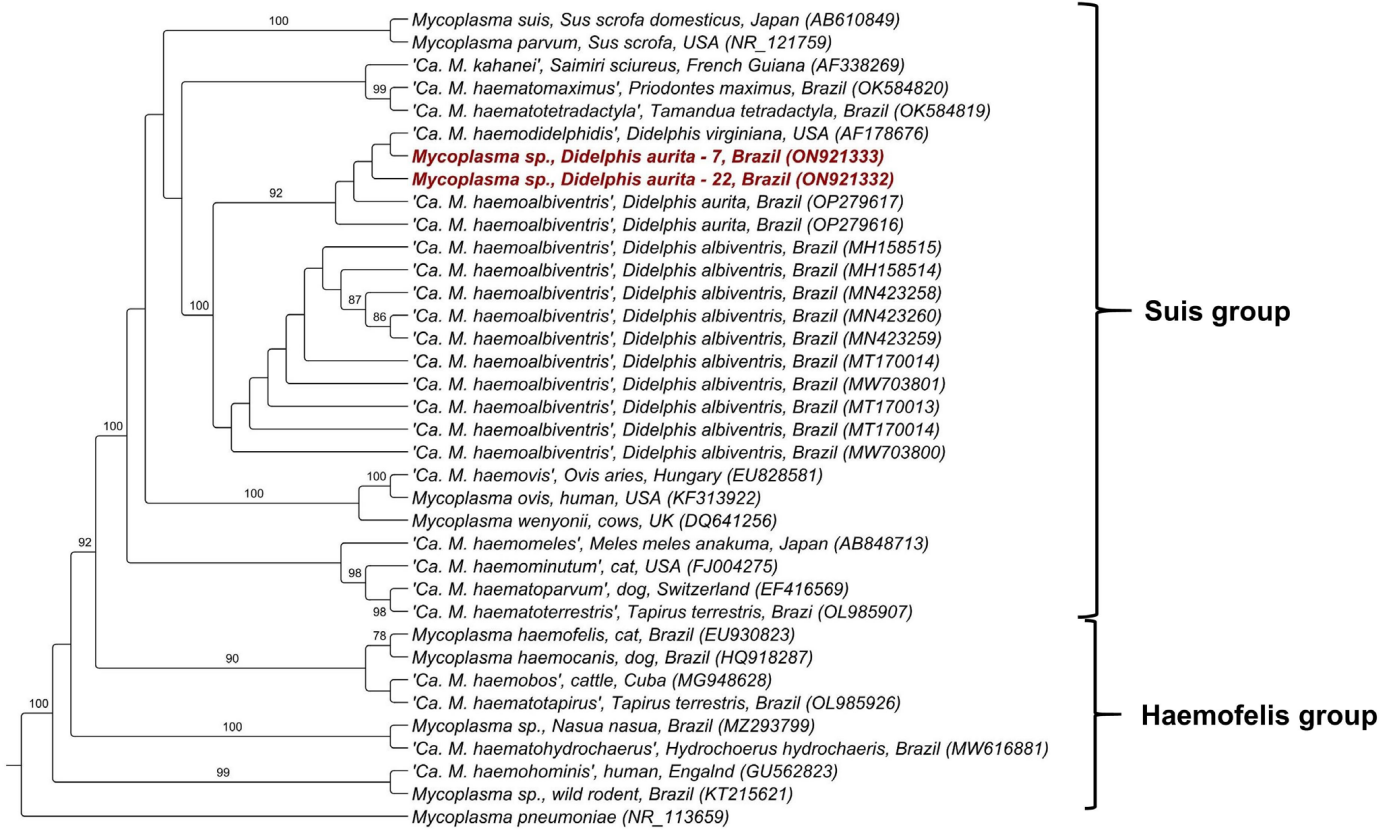
Phylogenetic tree based on an alignment of 841 bp of *Mycoplasma* spp. 16S rRNA sequences, using Maximum Likelihood method and TVMe+I+G evolutionary model. Sequences from the present study were highlighted in red *Mycoplasma pneumonie* was used as an outgroup.

The phylogenetic analysis inferred by Maximum Likelihood (ML) method and TVMe+I+G evolutionary model based on an alingment of 841 pb the 16S rRNA gene positioned the sequences obtained from *D. aurita* in a separate clade from the others, together with sequences previously detected in opossums. This clade was positioned within the *Mycoplasma suis* group and was subdivided into two sub-clades: while the first one comprised ‘*Candidatus* Mycoplasma haemodidelphidis’ previously detected in the Virginia opossum (*Didelphis virginiana*) from USA together with four sequences obtained in *D. aurita* from Brazil (two detected in the present study and two detected in opossums from Minas Gerais), with a bootstrap of 92%, the second sub-clade comprised ‘*Ca.* M. haemoalbiventris previously detected in *D. albiventris* from central-western and southern Brazil, with a bootstrap of 60% (Figure 1).

**Figure 2:**
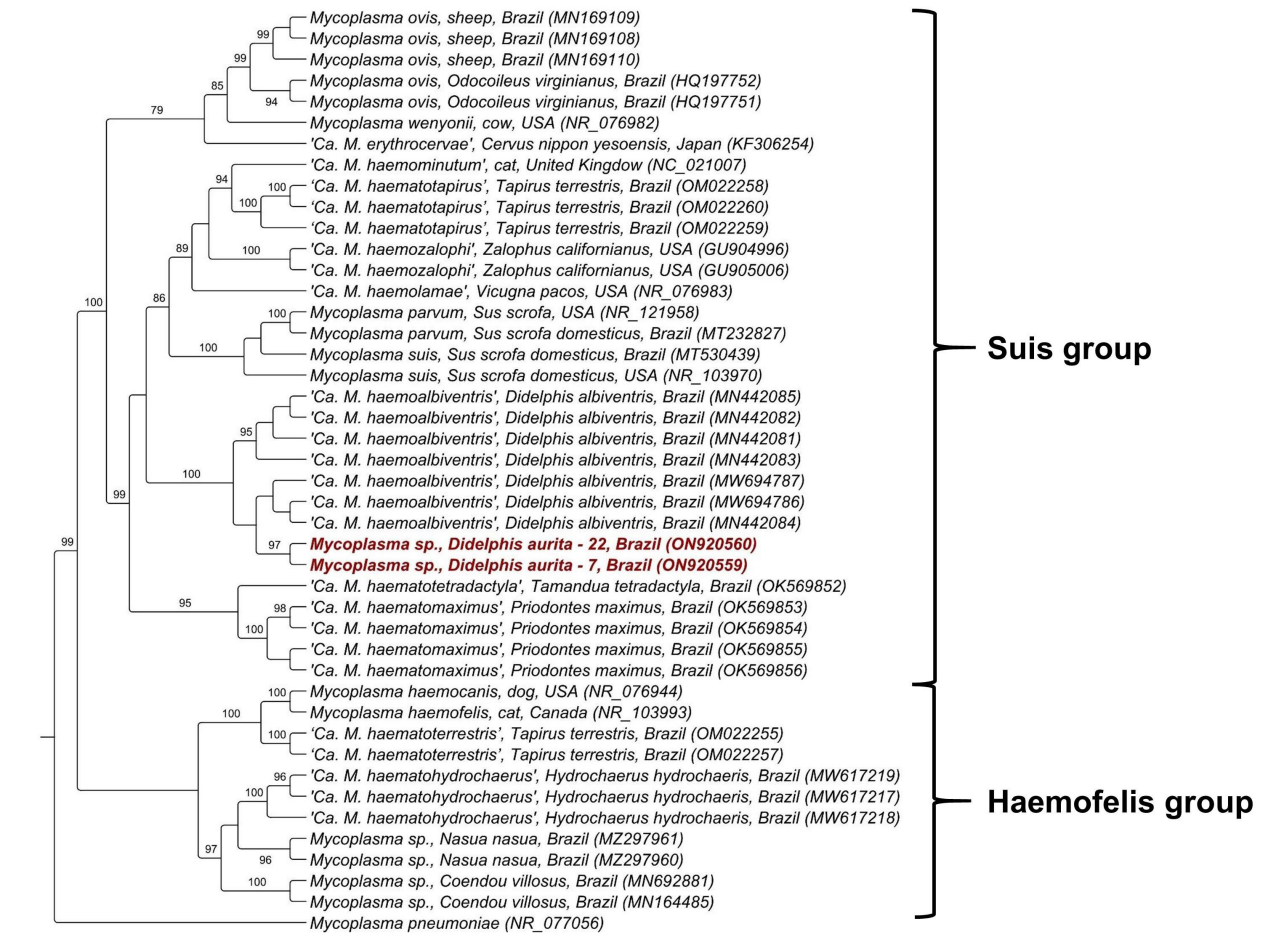
Phylogenetic tree based on an alignment of 634 bp of *Mycoplasma* spp. 23S rRNA sequences, using Maximum Likelihood method and TN+G as evolutionary model. Sequences from the present study were highlighted in red. *Mycoplasma pneumonie* was used as an outgroup.

The phylogenetic analysis inferred by ML method and TN+G evolutionary model based on an alingment of 634 pb the 23S rRNA gene positioned the two sequences detected herein into the ‘*Ca*. Mycoplasma haemoalbiventris’ clade, with a bootstrap of 100%. Unfortunately, ‘*Candidatus* Mycoplasma haemodidelphidis’ 23S rRNA sequences were not available in Genbank database until the moment the present study was written, precluding phylogenetic relatedness between hemoplasma 23S rRNA sequences detected in black-eared opossums with the *D. virginiana*-associated hemoplasma from the USA (Figure 2).

**Figure 3:**
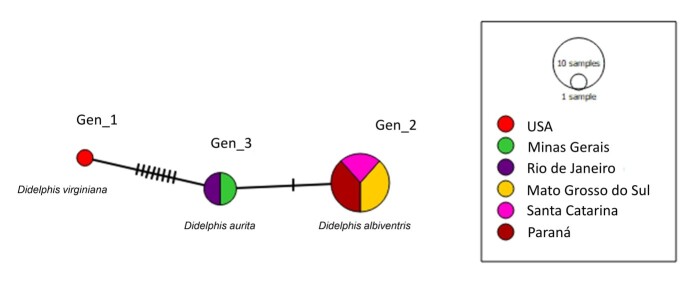
TCS network of 16S rRNA sequences performed with PopART v.1.7. software. The size of the circles varies according to the number of sequences belonging to each genotype and each color represents the location where each sequence was detected.

The genotype analysis based on the alignment of 18 hemotropic *Mycoplasma* sp. 16S rRNA sequences detected in opossums from Brazil and USA indicated the presence of three genotypes with nucleotide diversity (π)= 0.00162, haplotype diversity (dh) = 0.307, number of variable sites (VS) = 9, the average number of nucleotide differences (K) = 1.18301 and percentage of G+C content = 54.5%. The two sequences detected in the black-eared opossums sampled in the present study comprised a single genotype together with the two sequences obtained in opossums of the same species in Minas Gerais (Genotype #3). While genotype #1 comprised only the ‘*Ca.* Mycoplasma haemodidelphidis’ sequence detected in the USA and differed from genotype #3 from several mutational events, the genotype #2 comprised 15 ‘*Ca.* Mycoplasma haemoalbiventris’ sequences detected in *D. albiventris* from the Brazilian states of Paraná, Santa Catarina and Mato Grosso do Sul and differed from genotype #3 from a single mutational event (Figure 3).

## Discussion

In the present study, infection with hemotropic *Mycoplasma* spp. was found in 20% of the sampled black-eared opossums. Previous studies have found higher hemoplasma prevalence rates (ranging from 32.5-87.5%) in white-eared opossums from different geographic regions and biomes (Atlantic Forest and Cerrado) of Brazil (Massini et al., 2019; Gonçalves et al., 2020; Orozco et al., 2022; Pontarolo et al., 2021). Differences in environment conditions may influence habits and behavior of the opossums, and threfore, their exposure to hemoplasmas, which may explain the low percentage of positive animals found herein.

Although opossums of this study were rescued from the same Brazilian biome, there is great variation in the microenvironments where both wild animal screening centers are located. Such variations might have favored the occurrence of hemoplasmas, justifying the positivity observed only in animals rescued from CETAS-RJ, which is surrounded by the Atlantic Forest and not so closely located to urban areas. In fact, differences in the prevalence of hemotropic mycoplasmas in other species can be attributed to several factors, such as climate and habitats (Pontarolo et al., 2021). Additionally, a previous study has shown that raccoons and guignas from undisturbed habitats were more likely to be infected by hemoplasmas than animals from urban areas (Volokhov et al., 2017; Sacristán et al., 2019).

During the inspection of the opossums, ticks were not found parasitizing the sampled animals. On the other hand, hemoplasma positive white-eared opossums from the states of Paraná (Massini et al., 2019) and Mato Grosso do Sul (Gonçalves et al., 2020) were infested by *Amblyomma dubitatum* ticks. However, hemotropic *Mycoplasma* spp. DNA were not found in *A. dubitatum* ticks infesting ‘*Ca.* M. haemoalbiventris’-positive opossums from midwestern Brazil (Gonçalves et al., 2020). Recently, a high occurrence of ‘*Ca.* M. haemoalbiventris’ was found in *D. aurita* from Minas Gerais State, although infection was also not associated with the presence of ectoparasites (Orozco et al., 2022). Herein, *Ctenocephalides felis felis* fleas were found parasitizing three animals, although they were not PCR positive for hemotropic *Mycoplasma* spp. Previous studies have pointed out that white-eared opossums are frequently parasitized by *C. felis fleas* (Nascimento & Horta, 2014; Pontarolo et al., 2021), although there has not been enough evidence to support the hypotheses that hemotropic mycoplasmas are truly vector-borne pathogens to date. Indeed, Alcantara et al. (2022) did not find association between hemoplasmas, bats and ectoparasites by Multilayer network analyses, suggesting that hemoplasmas may be transmitted by alternative routes yet to be elucidated.

Phylogenetic analysis based on the 16S rRNA gene sequences showed that the hemoplasma genotype detected in the opossums was positioned between '*Ca.* Mycoplasma haemodidelphidis' detected in *D. virginiana* from North America and ‘*Ca.* Mycoplasma haemoalbiventris’ recently detected in *D. aurita* in the state of Minas Gerais, southeastern Brazil. Interestingly, the genotype network analysis showed that *D. aurita*-associated hemoplasma genotypes were shared by black-eared opossums from Minas Gerais and Rio de Janeiro. Neotropical black-eared opossums comprise two allopatric species*: D. aurita* and *D. marsupialis* (Cerqueira & Lemos, 2000). Both species tend to inhabit low-altitude regions with more preserved forest areas, which could hypothetically indicate a circulation of other hemoplasma species closely related in these individuals. Future studies aiming at investigating the genotype diversity of hemoplasma in *D. aurita, D. marsupialis* and *D. albiventris* from different Brazilian geographic regions will contribute to the understanding of the genetic diversity of this group of bacteria in marsupials from Brazil. A possible co-evolution between hemoplasmas and different Didelphidae is yet to be elucidated.

Although 66.6% of the black-eared opossums positive for hemoplasmas in the present study presented traumatic lesions, such finding is non-specific and must be related to their exposure to urban environments. However, Barker et al. (2010) suggests increased prevalence of hemoplasma in Tosas (Japanese fighting dogs) due to ingestion of infected blood during aggressive contact. The anemia detected in all positive opossums is probably associated to other causes, such as inflammatory or chronic conditions, as all *D. aurita* of the present study presented such hematological finding. Platelet and plasma protein values ​​found in hemoplasma positive animals were within the reference value, corroborating with Orozco et al. (2022). The white blood cell count of these animals revealed leukocytosis, a finding closely related to inflammatory conditions or systemic stress mediated by cortisol (Herrero-Cervera et al., 2022). Indeed, clinical alterations in the sampled animals were detected. Reports correlating concomitant diseases or situations of immunosuppression with the occurrence of infections by hemotropic mycoplasmas have already been described mainly in dogs and cats (Messick, 2004). To date, there are no reports in the literature that correlate the occurrence of infections by hemotropic *Mycoplasma* spp. with the presence of concomitant diseases in *Didelphis* sp. As so, whether infection by these agents may cause or predispose clinical disease in *D. aurita* remains to be fully established.

## Conclusion

Phylogenetic analysis of the 16S rRNA gene sequences showed that the hemoplasma detected in the black-eared opossums’ blood samples from Rio de Janeiro was positioned between *'Ca.* Mycoplasma haemodidelphis' detected in *D. virginiana* from North American and hemoplasmas recently detected in *D. aurita* from the state of Minas Gerais, Brazil. According to the Genotype Network Analysis, *D. aurita* specimes from the states of Minas Gerais and Rio de Janeiro share the same hemoplasma 16S rRNA genotype. Whole genome sequencing of opossums-associated hemoplasmas should be conducted aiming at shedding light in the real identity and species definition of hemotropic-*Mycoplasma* spp. infecting marsupials.
